# Impact of Conductive Yarns on an Embroidery Textile Moisture Sensor †

**DOI:** 10.3390/s19051004

**Published:** 2019-02-27

**Authors:** Marc Martínez-Estrada, Bahareh Moradi, Raúl Fernández-Garcia, Ignacio Gil

**Affiliations:** Department of Electronic Engineering, Universitat Politècnica de Catalunya, 08222 Terrassa, Spain; marc.martinez.estrada@upc.edu (M.M.-E.); bahareh.moradi@upc.edu (B.M.); ignasi.gil@upc.edu (I.G.)

**Keywords:** sensor, e-textile, embroidery, moisture, capacitive

## Abstract

In this work, two embroidered textile moisture sensors are characterized with three different conductive yarns. The sensors are based on a capacitive interdigitated structure embroidered on a cotton substrate with an embroidered conductor yarn. The performance comparison of three different type of conductive yarns has been addressed. In order to evaluate the sensor sensitivity, the impedance of the sensor has been measured by means of an LCR meter from 20 Hz to 20 kHz on a climatic chamber with a sweep of the relative humidity from 30% to 65% at 20 °C. The experimental results show a clear and controllable dependence of the sensor impedance with the relative humidity and the chosen conductor yarns. This dependence points out the optimum conductive yarn to be used to develop wearable applications for moisture measurement.

## 1. Introduction

Nowadays, there is a huge demand on the research and development of wearable sensors for biological sensing applications [1,2] like health monitoring, physical training [3], emergency rescue service and law-enforcement [4]. In order to develop these sensors, fabric substrates have been revealed as a natural and convenient choice in the development of wearable electronic applications due to the fact that humans have been covering their body with fabrics for thousands of years. The integration of these sensors over textiles can be carried out by means of several techniques, such as inkjet printing, screen printing, stamp transfer, electrospinning and dip coating [5]. Among all the textile techniques, embroidery has been revealed as one of the most effective techniques to implement wearable sensors. This fact is due to the availability of the manufacturing technology (industrial embroidery machines), the efficient exploitation of the expensive specialized conductive threads and the repeatability of the involved geometries and layouts [6].

The development of moisture sensors for wearable applications over textiles and outfits is a current research topic. This field has been investigated using different methodologies such as carbon nanotubes (CNT) [7], ink-jet technology [8], knitted fabric [9], screen printing [10] and embroidery [10,11]. Most of the papers in the literature are focused on moisture sensors over textiles for humidity ranges higher than 60% RH whereas no effective impact for moisture lower than 60% RH was observed [7,8]. In this paper, a comparison of the electrical properties of the embroidered sensor over cotton substrate with several types of conductive yarns are analysed and assessed in the range of 30% RH to 65% RH. This is a practical moisture range from the point of view of the human body and it has not been deeply investigated in the literature. In addition, the full characterization and electrical modelling has been carried out.

The remainder of the paper is organized as follows. Section 2 describes the material and methods including the conductive yarns used, the textile sensor layout and its implementation as well as the measurement set-up. In Section 3, the experimental results are shown and discussed. Finally, in Section 4, the conclusions are summarized.

## 2. Materials and Methods

**Figure 1:**
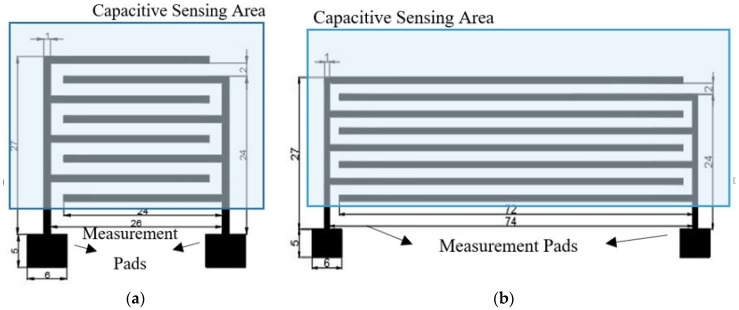
Layout and dimension detail of the proposed moisture sensor (in mm): (**a**) short sensor; (**b**) long sensor. The bottom squares correspond to the characterization pads.

**Figure 2:**
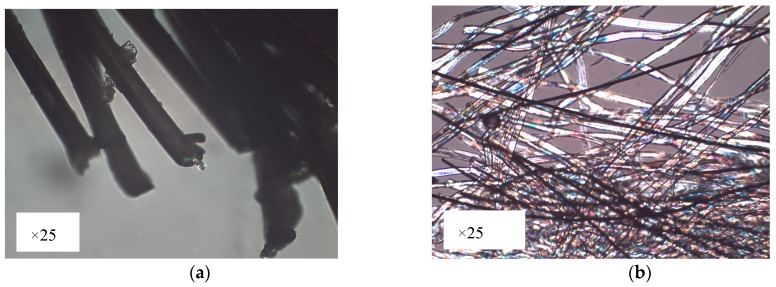
Microscope image of the used conductive yarns (**a**) Shieldex: this yarn is made of polyamide (PA) (inner) coated with pure silver (outer); (**b**) Bekaert: This yarn is made by mixing fibers (white fibers) of cotton with stainless steel (black fibers).

The proposed moisture sensor is based on a capacitive embroidered interdigitated structure whose dimensions are depicted in Figure 1. Three different conductive yarns are used to evaluate the behaviour of each yarn. Firstly, a commercial Shieldex 117/17 dtex 2-ply has been chosen. This yarn is made of polyamide (PA) coated with pure silver. Secondly, two commercial Bekaert yarn have been chosen. These yarns are made of stainless steel (SS) in mix with polyester (PES) or cotton (CO). One of the Bekaert yarns is made by polyester (80%) and stainless steel (20%). The other Bekaert yarn is made by a mix of cotton (80%) and stainless steel (20%) [12]. Furthermore, these yarns, Shieldex and Bekaert, are manufactured by using different techniques. Shieldex yarn is made by a coating of pure silver in the surface of the PA filament (Figure 2a). The Bekaert yarns are made by mixing fibers of different materials (cotton and polyester with stainless steel) at the beginning of the process to manufacture the yarn (Figure 2b). The most important properties for the aforementioned yarns are summarized in Table 1.

The considered conductive yarns are used in order to embroider the interdigitated structures on a high hygroscope substrate. In these cases, the permittivity of the substrate will be modified under the presence of water molecules. This mechanism allows achieving the sensing capability of the proposed devices. Specifically, a cotton substrate with a thickness (h) of 0.43 mm has been chosen. A Singer Futura XL-550 embroidery machine with a satin fill stitch pattern has been selected in order to achieve a homogeneous yarn distribution over the sensor surface.

**Figure 3:**
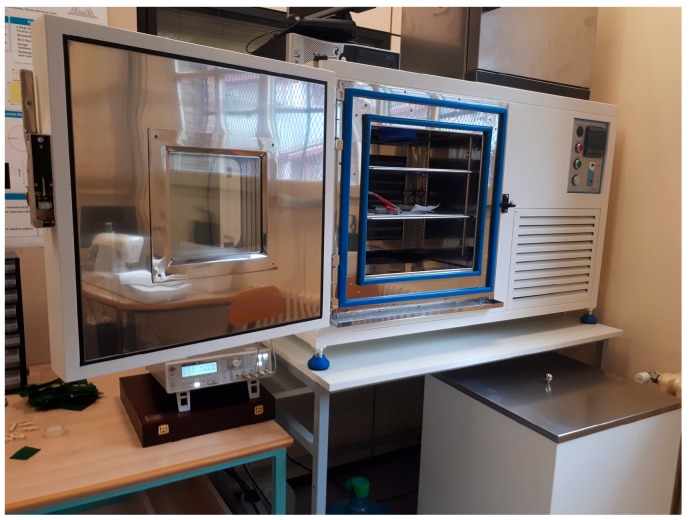
Image of the experimental setup.

In order to experimentally compare the sensors’ behaviour, the implemented devices have been tested in a CCK-25/48 Dycometal climatic chamber and the sensors impedances have been measured by means of an external Rohde & Schwarz HM8118 LCR meter. An image of the experimental setup is shown in Figure 3.

The sensors impedances have been measured from 20 Hz to 20 kHz in a 30% to 65% range of relative humidity environment, whereas the temperature has remained constant at 20 °C.

## 3. Results and Discussion

**Figure 4:**
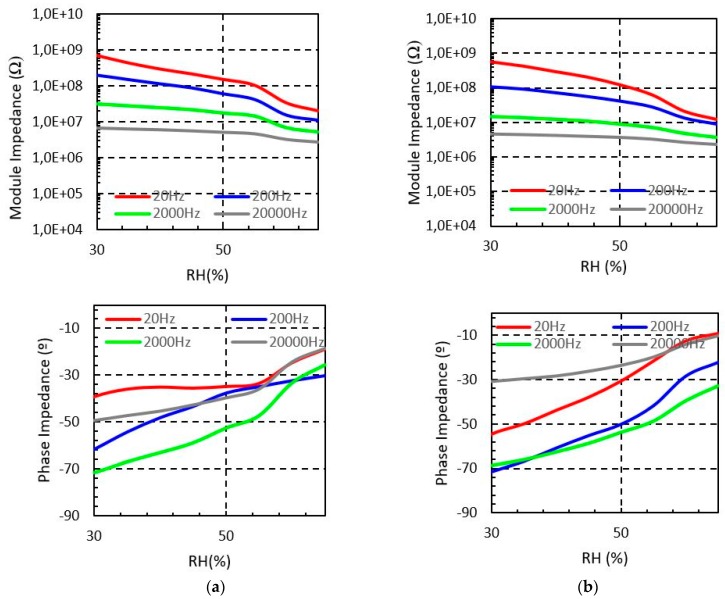
Measured Shieldex sensors impedance from 30% to 65% RH at different frequencies (T = 20 °C). (**a**) Short sensor impedance (**b**) Long sensor impedance.

Figure 4 shows the measured sensor impedance of the sensor embroidered with Shieldex when the moisture is swept from 30% to 65% for four different test frequencies. It is observed that the impedance module of the sensor is reduced when the environmental moisture increases, which confirms the functionality of the proposed structure as a moisture sensor. The measured phase impedance of the sensor denotes that for low relative humidity the sensor has a capacitive behaviour, as expected. Moreover, for higher relative humidity concentration the sensor tends to be resistive. The reason for this behaviour is the hydrophilic property of the cotton. Indeed, when the relative humidity increases, the cotton substrate absorbs water and the electrical permittivity of the substrate increases. As a result, the impedance of the sensor is reduced.

Long and short sensors show similar behaviour with the relative moisture. However, as it is expected, the impedance of the longer sensor (Figure 4b) is lower than the impedance of the short device (Figure 4a). In particular, for the 20 Hz test signal, the short sensor impedance decreases from 0.7 GΩ to 20.4 MΩ when the moisture increases from 30% to 65%, whereas, for the long sensor device it decreases from 0.57 GΩ to 12.5 MΩ for the same moisture range.

**Figure 5:**
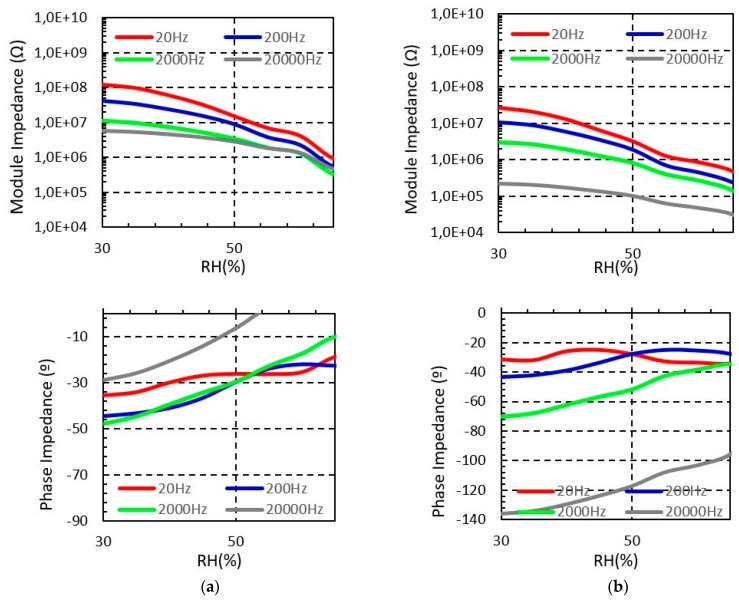
Measured Bekaert (PES-SS) sensors impedance from 30% to 65% RH at different frequencies (T = 20 °C). (**a**) Short sensor impedance (**b**) Long sensor impedance.

**Figure 6:**
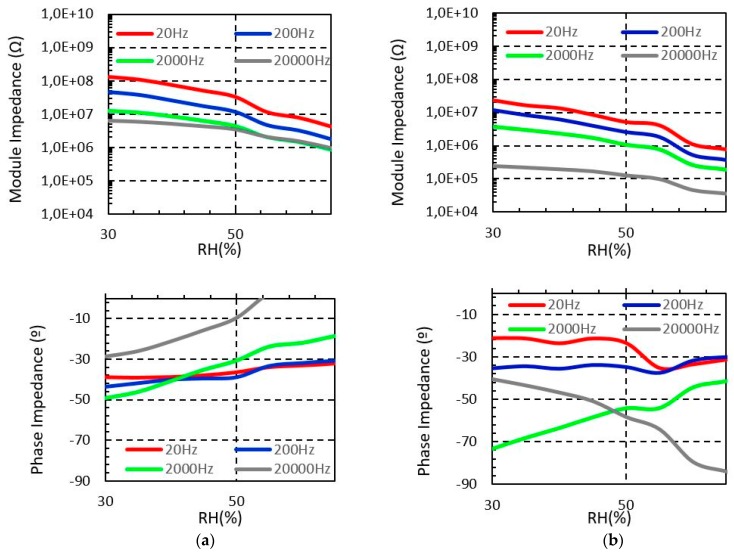
Measured Bekaert (CO-SS) sensors impedance from 30% to 65% RH at different frequencies (T = 20 °C). (**a**) Short sensor impedance (**b**) Long sensor impedance.

Figure 5 and Figure 6 show the measured impedance of the sensor embroidered with Bekaert PES-SS and Bekaert CO-SS, respectively. In all cases, it is observed that the sensor impedance module is reduced when the moisture increases, as we observed for the Shieldex yarn. However, both PES-SS and CO-SS show a significant impedance module reduction compared to Shieldex yarn, in all cases. In particular, for PES-SS at 20 Hz test, the impedance module decreases from 0.12 GΩ to 0.92 MΩ. In the case of the long sensor, the range decreases from 27 MΩ to 0.47 MΩ, whereas for CO-SS these values decrease from 0.13 GΩ to 4.2 MΩ and from 23.7 MΩ to 0.37 MΩ for the short and long sensor, respectively. The explanation for these differences between the Shieldex and Bekaert yarns is based on the electrical conductivity of the yarn, which depends on the conductive materials but also of the fabrication process. The non-conductive material of the yarn (i.e., polyester or cotton) does not have any significant impact on this behaviour.

It should be noticed that the obtained impedance values at the low frequency range are too high in order to develop a portable device based on the proposed sensor. These wearable devices are typically based on a single integrated circuit, such as the Texas Instrument AD5933 impedance converter [13]. Nevertheless, using the proposed sensor in the range of kHz allows obtaining impedances in the range of a MΩ. In these cases, the impedance values can be measured with those integrated circuits.

For comparison, in Table 2, the impedance range values at 2 kHz are summarized. Again, it is observed that for the same parameters of size, the sensors embroidered with Bekaert yarn had almost three times less module impedance than the Shieldex sensors. The difference should be due to the differences in the fabrication of each yarn. Bekaert yarns are made by fibres, whereas, Shieldex is made by coating a filament with silver. Another hypothesis consists of the fact that the Bekaert yarns can retain more moisture on their surface, and this moisture will decrease most effectively the values of impedance than in the other study case.

**Figure 7:**
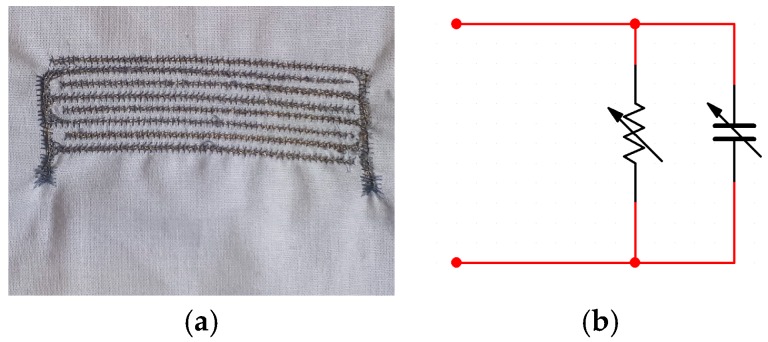
Proposed long sensor: (**a**) physical implementation and (**b**) electrical model.

If we focus on the electrical model according to the measured behaviour of the proposed sensors at 2 kHz, they can be modelled as a RC parallel lumped model (Figure 7b), where the R and C values are moisture dependent. The C represents the capacitance and R the current leakage of the interdigitated structure.

**Figure 8:**
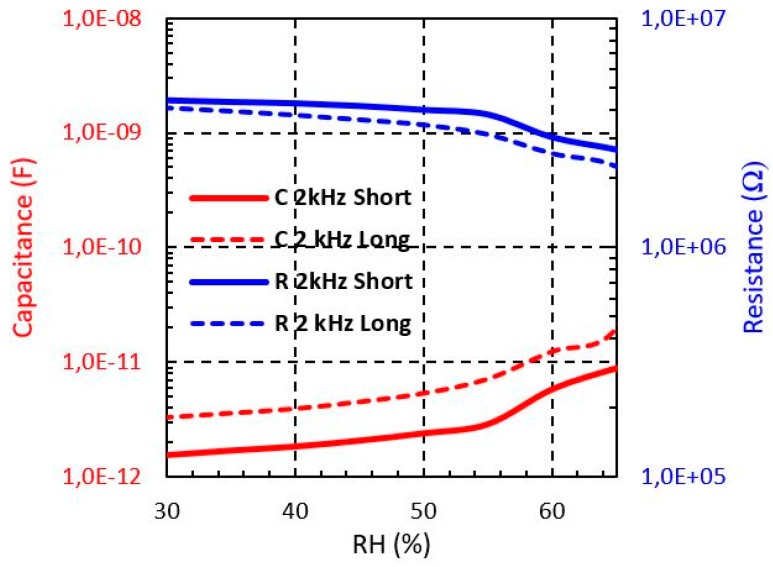
Measured Shieldex sensors impedance from 30% to 65% RH at different frequencies.

**Figure 9:**
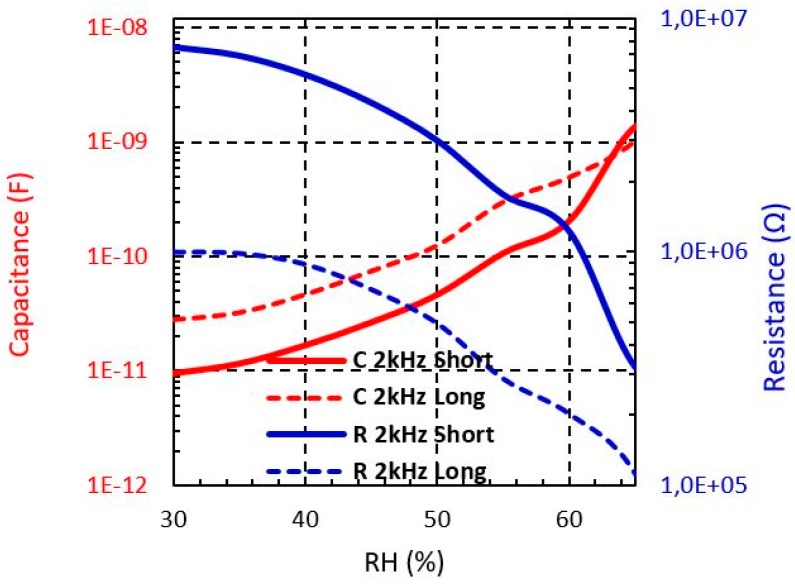
Measured Bekaert (PES-SS) sensors impedance from 30% to 65% RH at different frequencies.

**Figure 10:**
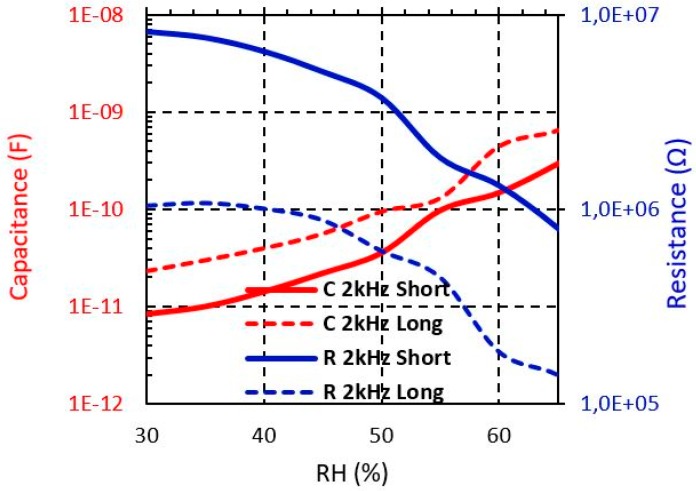
Measured Bekaert (CO-SS) sensors impedance from 30% to 65% RH at different frequencies.

From Figure 8, Figure 9 and Figure 10, the R and C dependence from 30% to 65% RH at 2 kHz is shown for both short and long sensors with Shieldex, Bekaert PES-SS and Bekaert CO-SS yarn, respectively. It can be observed that when the moisture level increases the capacitance is increased, whereas the resistance is reduced in all cases. It should be pointed out that the sensor based on Bekaert yarn shows a higher sensitivity with the moisture than the Shieldex yarn. This effect can be due to the moisture impact of the electrical properties of the yarn. Table 2 and Table 3 summarise the resistance and capacitance values of the electrical model when the moisture is swept from 30% to 65. The Bekaert yarns have a larger range of resistance value than the Shieldex sensors. However, between the Bekaert sensors it is not observed a relevant difference. Specifically, the resistance of Shieldex short sensor is reduced about 39%, whereas for Bekaert PES-SS and Bekaert CO-SS the reduction achieves 95.7% and 90.2%, respectively when the moisture is swept from 30% to 65% (Table 2). For the same moisture range, the capacitance increases about one order of magnitude (×10) for Shieldex yarn and about two order of magnitude (×100) for Bekaert PES-SS and Bekaert CO-SS (Table 3). This key fact points out that Bekaert yarns are more sensitive to develop moisture sensors, increasing the overall sensor sensitivity. It should be pointed out that for all conductive yarns and sensors a clear sensitivity change is produced around 55 % RH.

In the following Table 3 and Table 4 the values of resistance and capacitance for the studied sensors.

## 4. Conclusions

In this work, two interdigitated embroidered textile sensors have been proposed and characterized. The sensors have been embroidered over a cotton substrate with a commercial Shieldex 117/17 dtex 2 yarn, a commercial Bekaert (PES-SS) and Bekaert (CO-SS) 20/2 Tex. The measured results show that the sensor under analysis can be modelled by means of an RC parallel lumped circuit, where the R and C value are dependent on the moisture level. Particularly, a capacitance sensitivity at 2 kHz for short sensors of 0.21 pF/% RH, 38.87 pF/% RH and 8.05 pF/% RH is measured for Shieldex, PES-SS, CO-SS, respectively, whereas the resistance sensitivity is −49.7 kΩ/% RH, −207 kΩ/% RH and −212 kΩ/% RH. These results demonstrate experimentally the usefulness of the proposed sensors to achieve wearable moisture effective sensors for human body monitoring. In addition, the study points out that the Bekaert yarn PES-SS is preferred to increase the moisture sensitivity performance.

## Figures and Tables

**Table 1:** Most important properties about the yarns.

Properties	Shieldex	Bekaert (PES-SS)	Bekaert (CO-SS)
Density(tex)	11.7/2	20/2	20/2
Linear resistance (Ω/cm)	<30	50	35–70
Thread type	Twisted Multifilament	Ring yarn	Ring yarn

**Table 2:** Measured sensors module impedances ranges at 2 kHz.

Sensor	Shieldex	Bekaert (PES-SS)	Bekaert (CO-SS)
Short	31.2-5.18 MΩ	11.3-0.32 MΩ	12.6-0.85 MΩ
Long	14.8-3.71 MΩ	2.98-0.14 MΩ	3.62-0.19 MΩ

**Table 3:** Measured sensors resistance ranges at 2 kHz.

Sensor	Shieldex	Bekaert (PES-SS)	Bekaert (CO-SS)
Short	4.42-2.68 MΩ	7.57-0.32 MΩ	8.22-0.8 MΩ
Long	4.17-2.26 MΩ	0.99-0.11 MΩ	1.04-0.14 MΩ

**Table 4:** Measured sensors capacitance ranges at 2 kHz.

Sensor	Shieldex	Bekaert (PES-SS)	Bekaert (CO-SS)
Short	1.56-8.84 pF	9.48 pF-1.37 nF	8.37 pF-0.29 nF
Long	3.3-19.4 pF	28.3 pF-1.02 nF	23 pF-0.65 nF
